# Animal Models to Investigate the Pathogenesis of Rheumatic Heart Disease

**DOI:** 10.3389/fped.2014.00116

**Published:** 2014-11-04

**Authors:** Catherine M. Rush, Brenda L. Govan, Suchandan Sikder, Natasha L. Williams, Natkunam Ketheesan

**Affiliations:** ^1^Infectious Disease and Immunopathogenesis Research Group, Australian Institute of Tropical Health and Medicine, James Cook University, Townsville, QLD, Australia

**Keywords:** animal models, rheumatic fever, rheumatic heart disease, group A Streptococcus, molecular mimicry, autoimmune responses

## Abstract

Rheumatic fever (RF) and rheumatic heart disease (RHD) are sequelae of group A streptococcal (GAS) infection. Although an autoimmune process has long been considered to be responsible for the initiation of RF/RHD, it is only in the last few decades that the mechanisms involved in the pathogenesis of the inflammatory condition have been unraveled partly due to experimentation on animal models. RF/RHD is a uniquely human condition and modeling this disease in animals is challenging. Antibody and T cell responses to recombinant GAS M protein (rM) and the subsequent interactions with cardiac tissue have been predominantly investigated using a rat autoimmune valvulitis model. In Lewis rats immunized with rM, the development of hallmark histological features akin to RF/RHD, both in the myocardial and in valvular tissue have been reported, with the generation of heart tissue cross-reactive antibodies and T cells. Recently, a Lewis rat model of Sydenham’s chorea and related neuropsychiatric disorders has also been described. Rodent models are very useful for assessing disease mechanisms due to the availability of reagents to precisely determine sequential events following infection with GAS or post-challenge with specific proteins and or carbohydrate preparations from GAS. However, studies of cardiac function are more problematic in such models. In this review, a historical overview of animal models previously used and those that are currently available will be discussed in terms of their usefulness in modeling different aspects of the disease process. Ultimately, cardiologists, microbiologists, immunologists, and physiologists may have to resort to diverse models to investigate different aspects of RF/RHD.

## Introduction

Animal experimentation has been used for centuries to better understand human physiology and pathological processes, to improve diagnosis and to develop safe intervention, treatment, and prevention strategies. References to animal experimentation are found in the writings of the Greek philosopher–physician, Aristotle (384–322 BC) who was the first to carry out dissections. Later, Erasistratus (304–250 BC) was probably the first to perform experiments on living animals. However, it was Galen (AD 130–200) who justified animal experimentation as an arduous path to the truth, believing that assertions not based on experimentation do not lead to scientific progress (1). The twelfth century Arab physician, Ibn Zuhr (Avenzoar), introduced animal testing as an experimental technique for testing procedures before applying them to human patients (2). A valid animal model of a specific disease requires the process that initiates and perpetuates the pathological mechanisms to be identical or similar in the animal to that of the human condition that is being modeled.

Group A streptococci that trigger rheumatic fever (RF) and rheumatic heart disease (RHD) are uniquely human pathogens, with no other known natural host or environmental reservoir. Major shortcomings associated with research into RF/RHD, which may rely on studying human heart tissue from surgery or autopsy tissue from patients include heterogeneity and the ability to obtain sufficient numbers of high quality tissue specimens. Therefore, the availability of an animal model in which the pathogenic mechanisms responsible for RF/RHD could accurately be reproduced is crucial for furthering our understanding of the disease process, designing treatment and for testing efficacy, and safety of vaccine candidates against group A streptococci (GAS). Following the review of published literature on animal models for RF/RHD research in the last 85 years, we outline briefly the models that have been used to investigate the pathogenesis of RF/RHD. We outline the recent work on rodents and in particular rats, that have been instrumental in modeling some of the cardinal immunopathological features observed in RF/RHD.

## Early Experience with Animal Models

Developments in molecular biology, genomics, transgenic, and cloning techniques have enabled researchers to study human pathology in animals in greater depth. By investigating and identifying homologous genes across species, researchers can translate experimental data from animals to humans. The overwhelming majority of animals used in biomedical research are rodents such as mice and rats. These are ideal animal models because they are small, easy to handle, reproduce rapidly, have a relatively short life span and are relatively inexpensive to maintain in a laboratory setting. One of the major impediments in the field of RF/RHD research has been the lack of a universally accepted animal model. Furthermore animals are not easily infected by GAS: even when GAS infection is initiated, it is usually not sustained for extended periods of time.

Early experimental work to produce a suitable animal model of RF/RHD was based on the hypotheses that RF/RHD was caused either by persistent sub-clinical infection by GAS or by direct injury to cardiac tissue by GAS toxins. Therefore, most studies involved the introduction of whole bacteria or crude streptococcal preparations into various animals including mice, rats, guinea pigs (Table 1), rabbits, or non-human primates. While it was possible to observe myocardial necrosis, myocarditis, and endocarditis following immunization with GAS, none of the pathological lesions were considered representative of the hallmarks of RF/RHD, the development of Aschoff nodules and valvulitis. Animal models of RF/RHD developed prior to the 1970s paralleled the hypotheses for the causation of the disease process that were dominant at the time. When it became increasingly evident that the development of the inflammatory process observed in RF/RHD was immune-mediated, animal models were increasingly used to determine the role of host cross-reactive antibodies that developed post GAS infection. Although functional studies may be preferable in larger animals such as non-human primates, the prohibitive costs, and paucity of specific immunological reagents make such models less appropriate to determine the pathogenesis of RF/RHD.

**Table 1:** **Immunopathological changes in rodents investigated as models for rheumatic heart disease**.

Antigen (route of inoculation)	Histological changes	Antibody response	T cell response cytokine production	Cross-reactivity	References
**RATS (*Rattus norvegicus*)**					
Whole GAS (FP, SC)	**Myocarditis, valvulitis** lymphocyte, monocyte, MØ, giant cell, Aschoff-like cell, fibroblast	Anti-myocardial IgG	NA	Valvular protein, myocardial protein	Cavelti (3), Huang et al (4), Xie et al (5)
Recombinant proteins or peptides of GAS (SC, IP, FP)	**Myocarditis, valvulitis** T cell, MNC, neutrophil, Anitschkow-like cell	Anti-GAS IgG	CD3^+^, CD4^+^, CD8^+^, CD68^+^, TCR-αβ^+^	Cardiac myosin	Quinn et al (6), Lymbury et al (7), Gorton et al (8), Gorton et al (9), Kirvan et al (10)
**MICE (*Mus musculus*)**					
Cell wall fragments of GAS (IP)	**Myocarditis** MNC, PMNC, giant cell, Anitschkow-like cell	Anti-GAS IgG	NA	NA	Ohanian et al (11)
Recombinant protein of GAS (IP)	NA	Collagen IV reactive IgG	NA	Basement membrane collagen	Dinkla et al (12)
**GUINEA PIG (*Cavia porcellus*)**					
Whole GAS (IP, IV)	**Myocarditis, valvulitis** MNC	NA	NA	NA	Gross et al (13)
Cell wall fragments of GAS (FP)	NA	Anti-GAS IgG	NA	Cardiac sarcolemmal membrane	Yang et al (14)

*NA, not assessed; IV, intra-venous; SC, sub-cutaneous; IP, intra-peritoneal; FP, foot pad; MNC, mononuclear cell; PMNC, polymorphonuclear cell; MØ, macrophage; Ig, immunoglobulin; CD, cluster of differentiation; IFN, interferon; TCR, T cell receptor*.

## Current Concepts of Pathogenesis of Rheumatic Heart Disease

The pathogenesis of RF/RHD involves three principal elements: an infection caused by a specific strain of GAS, a susceptible host, and an aberrant immune response against GAS antigens that cross-react with host tissue (15, 16). The manifestations of RF/RHD are due to inflammatory changes that occur in cardiac tissue, joints, brain, blood vessels, and skin. Rheumatic carditis is the most serious consequence of the disease process while migratory polyarthritis and the neurologic manifestation Sydenham chorea (SC) may present in conjunction with carditis. Other clinical signs include the development of erythema marginatum and subcutaneous nodules (16). Host–GAS interactions initiating the immune responses cause carditis with subsequent GAS infections potentiating the disease process resulting in a cascade of events that cause hemodynamic changes culminating in irreversible cardiac damage and decompensatory cardiac failure.

Monoclonal antibodies derived from RF/RHD patients have provided evidence for cross-reactive autoantibodies that target GAS epitopes such as group A carbohydrate, *N*-acetyl-beta-d-glucosamine (GlcNAc), and M protein with host proteins including heart valve endothelium, laminin, and laminar basement membrane and cardiac myosin [Reviewed in Ref. (17, 18)]. GAS M proteins have been studies extensively and it is one of the important GAS proteins involved in inducing the autoimmune process. Over 220 different variants of the M protein have been described (19). While identification of M proteins is useful for epidemiological and vaccine studies not all GAS M types are implicated in the development of RF/RHD. Peripheral blood and heart-infiltrating T cells from patients with RF/RHD have revealed cross-reactivity of GAS M protein specific T cells with cardiac myosin. Experimental and clinical evidence suggests that antibodies interact with valvular endothelium and activate adhesion molecules with subsequent extravasation of T cells through the activated endothelium into the valve leading to the formation of granulomatous lesions. The inflammatory process that is thus triggered by antibodies to GAS infection activates adhesion molecules such as VCAM and ICAM, which play a role in the migration of leukocytes into the valvular tissue (17, 18). It has been demonstrated in patients with RF/RHD that specific chemokine genes, such as CXCL3 in the myocardium and CCL1 and CXCL9 in valvular tissue are upregulated (20). These chemokines could potentially mediate T cell recruitment to areas of inflammation. The specific cytokines produced by peripheral and heart-infiltrating T cells in patients have been identified (21) suggesting that Th1 and Th17 cells are involved in the development of carditis. Such cytokine profiles have also been observed in other autoimmune inflammatory conditions (22). Additionally the low levels of IL-4 produced by T cells infiltrating the valvular tissue may potentially contribute to the progression of valvular pathology (21).

Repeated GAS infection also leads to neovascularization within heart valves resulting in increased mononuclear cell infiltration resulting in cardiac and valvular damage. This process may also involve autoantibodies generated via epitope spreading following immune recognition of other components of the cardiac tissue such as vimentin and collagen released during tissue destruction (17).

One of the major manifestations associated with RF/RHD is SC. It has been found that antibodies from SC patients target the GAS carbohydrate epitope GlcNAc and react with gangliosides and dopamine receptors found on the surface of neuronal cells in the brain. These antibodies have been found to activate calcium calmodulin-dependent protein kinase II in neuronal cells and recognize the intracellular tubulin (23).

Molecular mimicry between GAS antigens and host antigens has been shown to initiate and potentiates the development of the clinical manifestations observed in RF/RHD. GAS have a variety of antigens and superantigens that are able to stimulate robust B and T cell responses to autoantigens (17, 18). Both human and animal studies have provided evidence that have been useful in formulating the potential mechanisms that lead to the post-infectious autoimmune sequelae. Animal models of RF/RHD and SC have been important in characterizing mimicry in carditis and SC as well as attempting to identify the pathogenic epitopes of the autoantigens and GAS antigens involved in the pathogenesis of RF/RHD. However, several facets of the pathological process at a cellular and molecular level that initiate inflammatory changes in the different organs and tissue in RF/RHD remain undefined. Fundamental concepts including which GAS strains and what combination of GAS antigens are involved in the breakdown of tolerance during the disease process are unclear. Studies to address some of these fundamental issues require the use of well characterized animal models that encompass the features observed in RF/RHD.

## Use of Rodent Models to Determine the Autoimmune Process

**Figure 1:**
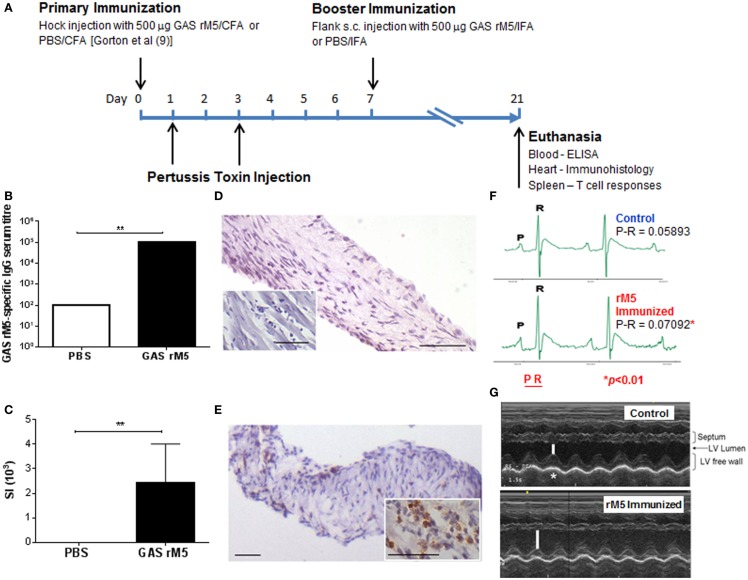
**Immunization protocol for the induction of autoimmune valvulitis in Lewis rats and the immunological, histological, and functional changes following immunization with recombinant streptococcal M protein**. **(A)** The induction of valvulitis in the rat autoimmune valvulitis (RAV) model of RF/RHD involves a primary immunization of female Lewis rats (under isoflurane anesthesia) with 500 μg GAS rM5 protein (or PBS as a negative control) in complete Freund’s adjuvant (CFA) administered subcutaneously (s.c.) in the hock on day 0. On days 1 and 3, rats are injected intraperitoneally (i.p.) with an additional adjuvant being either 0.3 μg commercially purchased pertussis toxin [PTx; (7)] or 10^10^ CFU formalin-killed *Bordetella pertussis* (6, 8, 9) each in 200 μl PBS. On day 7, rats receive a booster immunization with 500 μg GAS rM5 protein (or PBS as a negative control) in incomplete Freund’s adjuvant (IFA) administered s.c. in the flank under anesthesia. On day 21, the rats are euthansed by CO_2_ asphyxiation to harvest blood and organs for histological examination of heart tissue and determination of GAS rM5-specific antibody levels and assessment of T cell function. **(B)** GAS rM5-specific IgG antibodies in rat (*n* = 5) serum were detected by ELISA. The highest serum dilution that was positive for GAS rM5-specific IgG antibodies (cut-off value 3 SD higher than the mean for the known negative control serum) was recorded as the serum titer. Serum from rats immunized with GAS rM5 contained significantly higher GAS rM5-specific antibodies compared to control (*P* = 0.007). **(C)** Proliferative response of GAS rM5-specific T cells from spleens of rats (*n* = 5) was determined by ^3^H-thymidine incorporation assay and found to be significantly higher than in controls immunized with PBS (*P* = 0.009). Bars depict the mean ± SEM. **P ≤ 0.01. Immunohistological changes **(D,E)** in representative valvular tissue and myocardium (inset) from **(D)** controls and **(E)** rM5-immunized animals. Immunohistochemical staining of infiltrating mononuclear cells demonstrates the presence of CD4+ T cells in rM5-immunized animals compared to controls. Scale bar indicates 50 μM (DAB staining). **(F)** ECG complexes from a control and a rM5-immunized rat demonstrate significantly longer P-R interval in the rM5-immunized rats (Images courtesy of Dr Lisa Chilton, James Cook University). **(G)** Echocardiographs from control and an rM5-immunized rat demonstrate reduction in fractional shortening due to reduced LV contractility. Bars represent the width of the left ventricle (LV) chamber during contraction (*Images courtesy of Drs Lisa Chilton and Jane Day, James Cook University).

To further investigate the pathogenesis of RF/RHD and in particular to determine the contribution of autoreactive B and T cells in the RF/RHD pathogenesis, suitable animal models were investigated. Huber and Cunningham (24) produced myocarditis in MRL+/+ mice immunized with specific N-terminal peptides of GAS M5. These studies were one of the first to demonstrate that mimicry between a pathogen derived epitope and a host protein could break tolerance and trigger an autoimmune process in a susceptible host. The same group at the Oklahoma University Health Sciences Center subsequently developed a more robust animal model for RF/RHD (6, 25). Female Lewis rats immunized with GAS M protein (Figure 1A) exhibit myocardial lesions similar to those observed in patients with RF/RHD. More importantly, valvular pathology was observed for the first time using this model (5–8, 10, 25). This model is discussed in more detail below.

In recent years extensive work on murine models have been conducted to determine the efficacy of anti-GAS vaccine candidates in terms of their ability to protect against GAS infections (18, 26, 27). These murine models have been used mostly if not exclusively to determine vaccine efficacy. Although an HLA class II transgenic mouse model has been used to investigate both the protective immune responses induced by a vaccine candidate and determine whether it induces histological changes in host tissue (26), this model may not be very appropriate for the purpose of determining cross-reactivity in host tissue. A year following immunization with the candidate vaccine preparation the investigators did not observe any histological changes in various organs in the immunized mice. Although the vaccine preparation was considered to be safe, control transgenic mice developed additional complications. These complications may deter researchers from using such a model to investigate tissue cross-reactivity and vaccine safety.

## Rat Autoimmune Valvulitis Model of Rheumatic Heart Disease

The Lewis rat model of RF/RHD developed in the late 90s (6), exhibited myocardial lesions similar to those observed in RF/RHD following immunization with GAS M6 protein (Figure 1). More importantly, this Lewis rat model is the first in which valvular changes akin to human pathology has been demonstrated. Valvular lesions observed at the valve surface endothelium spread into the valve. Anitschkow cells and verruca-like lesions have also been observed. Due to the valvular involvement observed in immunized animals, this model has been referred to as the rat autoimmune valvulitis (RAV) model (8). T cells from recombinant M6-immunized (rM6) rats proliferated *in vitro* in the presence of cardiac myosin. In addition a T cell line produced from GAS rM6-immunized rats proliferated in the presence of cardiac myosin and GAS rM6 protein. When Galvin and colleagues (25) co-cultures myosin-sensitized lymphocytes isolated from the hearts of Lewis rats with peptides of GAS M5 protein, heart-infiltrating lymphocytes proliferated in response to peptides within the B-repeat region of the GAS M protein. Their work provided evidence that an immune response against cardiac myosin could potentially lead to valvular heart disease and the infiltration of the heart by GAS M protein-reactive T cells.

Using the same protocol to initiate valvulitis, Lymbury et al. (7) demonstrated that 80% of Lewis rats immunized with a pool of 15, 20-mer overlapping peptides spanning the conserved C-repeat region of the GAS M5 developed inflammatory lesions in both the myocardium and valvular tissue. These studies highlighted the role for GAS M protein-specific autoreactive T cells in the development of cardiac lesions. T cells from rats immunized with the conserved region peptides proliferated in response to the immunogen and to cardiac myosin. Further proof of the role of both humoral and cellular responses (Figures 1B–E) in the pathogenesis of RF/RHD was demonstrated by Gorton et al (8). It was found that GAS rM5 protein elicited opsonic antibodies in Lewis rats, which recognized epitopes within the B- and C-repeat regions of M5. A single peptide from the GAS M5 B-repeat region induced lymphocytes that responded to both recombinant M5 and cardiac myosin. Additionally, it was found that rats immunized with GAS rM5 protein developed valvular lesions (Figures 1D,E), distinguished by infiltration of CD3+, CD4+, and CD68+ cells into valve tissue, consistent with human studies. This suggests that RF/RHD is mediated by inflammatory responses involving both CD4+ T cells and macrophages. Recent proof of concept work undertaken by this group on the RAV model has also demonstrated that repetitive immunization with GAS rM5 increases both B and T cell sensitization leading to increased inflammatory cell infiltration that could potentially lead to severe cardiac damage. This observation further demonstrates that the immunopathology in the RAV model reflects the human condition, where repetitive GAS infections lead to exacerbation of RF/RHD, which culminates in cardiac failure.

The Lewis rat model has also been used to immunize with formalin-killed and sonicated GAS (5). The investigators were able to demonstrate in rats killed 12 weeks following immunization only 50% (4/8) developed myocarditis and valvulitis. In contrast, animals sacrificed 24 weeks following GAS immunization demonstrated myocardial and valvular damage and developed rheumatic-like myocarditis with 62.5% (5/8) developing chronic valvulitis. Histological manifestations of the hearts in this group demonstrated “Aschoff-like” cells, verrucous vegetation, and chronic lesions including fibrosis and neovascularization, hallmark of chronic rheumatic valvulitis.

To identify the epitopes of M5 protein that produce valvulitis, and to prove that M protein-specific T cells may be important mediators of valvulitis, Kirvan and colleagues (10) used synthetic peptides spanning all three repeat regions of GAS M5 (A, B, and C-repeat regions) contained within the extracellular domain of the streptococcal M5 protein to immunize Lewis rats. Peptides NT4, NT5/6, and NT7 from the A repeat region induced valvulitis similar to the pepsin fragment of M5 protein. T cell lines from rats with valvulitis also recognized peptides NT5/6 and NT6. They also conducted passive transfer of a NT5/6-specific T cell line into naïve rats, which produced valvulitis with characteristic CD4+ T cell infiltration and upregulation of VCAM demonstrating experimentally that M protein-specific T cells are important mediators of valvulitis.

To our knowledge the RAV model has not been widely used to investigate the safety of anti-GAS vaccine candidates by assessing their potential to initiate autoimmune pathology. However, prior to a recent human Phase 1 clinical trial for a GAS vaccine based on the J8 construct, the RAV model was used to test for safety of the vaccine preparation. None of the animals immunized with the vaccine preparation in the authors’ laboratory developed either carditis or valulitis. While the RAV model has been useful in characterizing key aspects involved in the autoimmune process in RF/RHD pathogenesis, its potential has not yet been fully realized. Extensive characterization of cardiac function in the RAV model (Figures 1F,G) may lead to greater acceptance of the RAV model among researchers working on different aspects of RF/RHD.

## Rat Model for Sydenham’s Chorea

One of the manifestations of RF/RHD is the development of the SC, which is a neurological disorder characterized by rapid, uncoordinated jerking movements affecting the face, hands, and feet. Although it is reported to occur in a quarter of patients with RF/RHD in some regions, it may also be the presenting symptom of RF/RHD (16). A team of researchers from Tel Aviv University and the Oklahoma University Health Sciences Center developed a model for SC using male Lewis rats, which were exposed to GAS antigen (28, 29). Following immunization with GAS, the rats exhibited neurological motor symptoms and compulsive behavior. These symptoms were alleviated by the D2 blocker haloperidol and the selective serotonin reuptake inhibitor paroxetine, medications that are used to treat motor symptoms and compulsions in GAS-related neuropsychiatric disorders. Recent studies published by these investigators revealed that antibodies purified from the sera of GAS-exposed rats and infused into the striatum of naïve rats led to behavioral and motor alterations mimicking those seen in GAS-exposed rats (29). IgG from GAS-exposed rats reacted with dopamine and serotonin receptors *in vitro*, demonstrating the potential pathogenic role of autoantibodies produced following exposure to GAS.

## Summary

There are many fundamental immunological questions that need to be answered if effective RF/RHD control strategies are to be implemented worldwide. These questions include: (1) the identity and dominant hierarchy of streptococcal antigens in driving immune-mediated cardiac pathology; (2) the precise cell-mediated and antibody mechanisms involved in disease initiation and progression; and (3) whether disease can be prevented by regulating or switching off pathogenic immune responses. Targeting key processes involved in disease mechanisms, i.e., regulation of destructive immune responses directed against the host, may provide adjunct management strategies for RF/RHD. The RAV model is currently the only model available that is suitable for such investigations.

## Conflict of Interest Statement

The authors declare that the research was conducted in the absence of any commercial or financial relationships that could be construed as a potential conflict of interest.

## References

[1] Loew FL Cohen BJ Laboratory animal medicine: historical perspective. In: Fox JG Anderson LC Loew FM Quimby FW, editors. Laboratory Animal Medicine. 2nd ed Academic Press (2002). p. 1–17.

[2] Hajar R Animal testing and medicine. Heart Views (2011) 12(1):42 10.4103/1995-705X.81548 21731811 PMC3123518

[3] Cavelti PA. Studies on the pathogenesis of rheumatic fever; cardiac lesions produced in rats by means of autoantibodies to heart and connective tissues. Arch Pathol (Chic) (1947) 44(1):13–27. 20258279

[4] Huang J Xie X Lin ZF Luo MQ Yu BY Gu JR. Induction of myocarditis lesions in Lewis rats by formalin-killed cells of group A *Streptococcus*. J Int Med Res (2009) 37(1):175–81. 10.1177/147323000903700121 19215688

[5] Xie X Zhou H Huang J Huang H Feng Z Mei K An animal model of chronic rheumatic valvulitis induced by formalin-killed *Streptococci*. Rheumatol Int (2010) 30(12):1621–5. 10.1007/s00296-009-1246-3 20012632

[6] Quinn A Kosanke S Fischetti VA Factor SM Cunningham MW. Induction of autoimmune valvular heart disease by recombinant streptococcal m protein. Infect Immun (2001) 69(6):4072–8. 10.1128/IAI.69.6.4072-4078.2001 11349078 PMC98471

[7] Lymbury RS Olive C Powell KA Good MF Hirst RG LaBrooy JT Induction of autoimmune valvulitis in Lewis rats following immunization with peptides from the conserved region of group A streptococcal M protein. J Autoimmun (2003) 20(3):211–7. 10.1016/S0896-8411(03)00026-X 12753806

[8] Gorton D Govan B Olive C Ketheesan N. B- and T-cell responses in group a *Streptococcus* M-protein- or peptide-induced experimental carditis. Infect Immun (2009) 77(5):2177–83. 10.1128/IAI.01514-08 19273562 PMC2681745

[9] Gorton D Blyth S Gorton JG Govan B Ketheesan N. An alternative technique for the induction of autoimmune valvulitis in a rat model of rheumatic heart disease. J Immunol Methods (2010) 355(1–2):80–5. 10.1016/j.jim.2010.02.013 20206182

[10] Kirvan CA Galvin JE Hilt S Kosanke S Cunningham MW. Identification of streptococcal m-protein cardiopathogenic epitopes in experimental autoimmune valvulitis. J Cardiovasc Transl Res (2014) 7(2):172–81. 10.1007/s12265-013-9526-4 24346820 PMC3943786

[11] Ohanian SH Schwab JH Cromartie WJ. Relation of rheumatic-like cardiac lesions of the mouse to localization of group A streptococcal cell walls. J Exp Med (1969) 129(1):37–49. 10.1084/jem.129.1.37 5782771 PMC2138592

[12] Dinkla K Rohde M Jansen WT Kaplan EL Chhatwal GS Talay SR. Rheumatic fever-associated *Streptococcus pyogenes* isolates aggregate collagen. J Clin Invest (2003) 111(12):1905–12. 10.1172/JCI17247 12813026 PMC161421

[13] Gross L Loewe L Eliasoph B Attempts to reproduce rheumatic fever in animals. J Exp Med (1929) 50(1):41–65 10.1084/jem.50.1.41 19869605 PMC2131601

[14] Yang LC Soprey PR Wittner MK Fox EN. Streptococcal-induced cell-mediated-immune destruction of cardiac myofibers in vitro. J Exp Med (1977) 146(2):344–60. 10.1084/jem.146.2.344 327015 PMC2180774

[15] Bryant PA Robins-Browne R Carapetis JR Curtis N. Some of the people, some of the time: susceptibility to acute rheumatic fever. Circulation (2009) 119(5):742–53. 10.1161/CIRCULATIONAHA.108.792135 19204317

[16] Chakravarty SD Zabriskie JB Gibofsky A. Acute rheumatic fever and *Streptococci*: the quintessential pathogenic trigger of autoimmunity. Clin Rheumatol (2014) 33(7):893–901. 10.1007/s10067-014-2698-8 24894108

[17] Cunningham MW *Streptococcus* and rheumatic fever. Curr Opin Rheumatol (2012) 24(4):408–16 10.1097/BOR.0b013e32835461d3 22617826 PMC3645882

[18] Guilherme L Kalil J. Rheumatic heart disease: molecules involved in valve tissue inflammation leading to the autoimmune process and anti-S. pyogenes vaccine. Front Immunol (2013) 4:352. 10.3389/fimmu.2013.00352 24198818 PMC3812567

[19] Sanderson-Smith M De Oliveira DM Guglielmini J McMillan DJ Vu T Holien JK A systematic and functional classification of *Streptococcus pyogenes* that serves as a new tool for molecular typing and vaccine development. J Infect Dis (2014) 210(8):1325–38. 10.1093/infdis/jiu260 24799598 PMC6083926

[20] Fae KC Palacios SA Nogueira LG Oshiro SE Demarchi LM Bilate AM CXCL9/Mig mediates T cells recruitment to valvular tissue lesions of chronic rheumatic heart disease patients. Inflammation (2013) 36(4):800–11. 10.1007/s10753-013-9606-2 23417848 PMC3708284

[21] Guilherme L Cury P Demarchi LM Coelho V Abel L Lopez AP Rheumatic heart disease: proinflammatory cytokines play a role in the progression and maintenance of valvular lesions. Am J Pathol (2004) 165(5):1583–91. 10.1016/S0002-9440(10)63415-3 15509528 PMC1618676

[22] Mills KH. TLR-dependent T cell activation in autoimmunity. Nat Rev Immunol (2011) 11(12):807–22. 10.1038/nri3095 22094985

[23] Kirvan CA Swedo SE Kurahara D Cunningham MW. Streptococcal mimicry and antibody-mediated cell signaling in the pathogenesis of Sydenham’s chorea. Autoimmunity (2006) 39(1):21–9. 10.1080/08916930500484757 16455579

[24] Huber SA Cunningham MW. Streptococcal M protein peptide with similarity to myosin induces CD4+ T cell-dependent myocarditis in MRL/++ mice and induces partial tolerance against coxsakieviral myocarditis. J Immunol (1996) 156(9):3528–34. 8617982

[25] Galvin JE Hemric ME Kosanke SD Factor SM Quinn A Cunningham MW. Induction of myocarditis and valvulitis in Lewis rats by different epitopes of cardiac myosin and its implications in rheumatic carditis. Am J Pathol (2002) 160(1):297–306. 10.1016/S0002-9440(10)64373-8 11786423 PMC1867128

[26] Guerino MT Postol E Demarchi LM Martins CO Mundel LR Kalil J HLA class II transgenic mice develop a safe and long lasting immune response against StreptInCor, an anti-group A *Streptococcus* vaccine candidate. Vaccine (2011) 29(46):8250–6. 10.1016/j.vaccine.2011.08.113 21907752

[27] Pandey M Wykes MN Hartas J Good MF Batzloff MR. Long-term antibody memory induced by synthetic peptide vaccination is protective against *Streptococcus pyogenes* infection and is independent of memory T cell help. J Immunol (2013) 190(6):2692–701. 10.4049/jimmunol.1202333 23401589 PMC3594626

[28] Brimberg L Benhar I Mascaro-Blanco A Alvarez K Lotan D Winter C Behavioral, pharmacological, and immunological abnormalities after streptococcal exposure: a novel rat model of Sydenham chorea and related neuropsychiatric disorders. Neuropsychopharmacology (2012) 37(9):2076–87. 10.1038/npp.2012.56 22534626 PMC3398718

[29] Lotan D Benhar I Alvarez K Mascaro-Blanco A Brimberg L Frenkel D Behavioral and neural effects of intra-striatal infusion of anti-streptococcal antibodies in rats. Brain Behav Immun (2014) 38:249–62. 10.1016/j.bbi.2014.02.009 24561489 PMC4000697

